# Preoperative absolute neutrophil count: a potential indicator for prognosis in carcinoembryonic antigen normal stage I non-small cell lung cancer

**DOI:** 10.3389/fonc.2025.1632597

**Published:** 2025-10-27

**Authors:** Bailin Wang, Long Liu, Meiqi Cui, Qianwen Ye, Panhua Li, Bing Yan

**Affiliations:** ^1^ Department of Thoracic Surgery, Hainan Hospital of Chinese People's Liberation Army (PLA) General Hospital, Sanya, Hainan, China; ^2^ Department of Traditional Chinese Medicine, Shanghai Tianyou Hospital, Shanghai, China; ^3^ Department of Intensive Care Unit, Hainan Hospital of Chinese People's Liberation Army (PLA) General Hospital, Sanya, Hainan, China; ^4^ Department of Oncology, Hainan Hospital of Chinese People's Liberation Army (PLA) General Hospital, Sanya, Hainan, China

**Keywords:** lung cancer, carcinoembryonic antigen, neutrophil count, disease-free survival, overall survival

## Abstract

**Background:**

Carcinoembryonic antigen (CEA) is still the most valuable tumor marker in the diagnosis and prognosis of non-small cell lung cancer (NSCLC) patients; however, its application is largely limited by its low sensitivity in stage I cases. Research on reliable and highly cost-effective prognostic indicators in CEA normal stage I NSCLC is still needed.

**Methods:**

A retrospective study was conducted in CEA normal stage I NSCLC patients. The prognostic value of peripheral blood cell fractions, including the absolute neutrophil count (ANC), was tested, and the differences in clinical features among the ANC-low or ANC-high subgroups were checked. The disease-free survival (DFS) and overall survival (OS) differences in these subgroups were run by Kaplan–Meier analysis, and the risk factors for survival were validated by a Cox proportional hazards model.

**Results:**

Among the tested peripheral blood cell fractions, only ANC was found to be a significant factor in predicting DFS (P = 0.011) and OS (P=0.043). The ANC displayed a positive correlation with other fractions, including the absolute lymphocyte count (R=0.26, P<0.001), absolute monocyte count (R=0.56, P<0.001), and platelet count (R=0.29, P<0.001). With a cutoff at 3879/mm^3^, 83.72% (252/301) of patients were divided into ANC-low and 16.28% (49/301) into ANC-high. Patients in the ANC-low group also presented a superior DFS (log rank=8.64, P = 0.003) and OS (log rank=9.86, P = 0.002) than those in the ANC-high group; however, the ANC level was not validated as an independent prognostic factor for both DFS and OS.

**Conclusion:**

Compared to other peripheral blood cell fractions, preoperative ANC was found to be a useful prognostic indicator in CEA normal stage I NSCLC; however, it was not validated as an independent prognostic factor and additional studies for its role in prognosis for these patients are still needed in future.

## Introduction

Lung cancer is still a heavy health and economic burden in China, with over 80% being non-small cell lung cancer (NSCLC) (1, 2), which mainly comprises adenocarcinoma (ADC) and squamous carcinoma (SCC). In recent decades, although overall survival (OS) for locally advanced NSCLC and those with remote lesions has greatly improved due to the success of immunotherapy-based regimens and targeted therapies (3–5), radical resection is still the optimal choice for early-stage cases, specifically stage I. Fortunately, with the success of clinical trials, including ADAURA (6), Keynote091 (7), and IMpower 010 (8), the survival for stage IB cases has been further guaranteed. Nonetheless, the 5-year OS for stage IA patients ranges from 77-92%, declining up to 68% for stage IB patients (9). Although many innovative prognostic indicators, such as molecular residual disease (MRD), have been reported and are very helpful in subsequent treatment strategy decisions in these cases (10, 11), searching for easily accessible and highly cost-effective prognostic indicators is still needed at present in practice.

Carcinoembryonic antigen (CEA) is a classic tumor marker in NSCLC (12) that plays an important role not only in diagnosis (13) but also in prognosis (14). However, the usefulness of CEA in practice was also blocked due to its relatively low sensitivity, particularly in stage I cases, as previous studies suggested a positive rate ranging from 16.22% (145/894 in stage IA (15)) to 33.62% [274/815 in stage I (16)]. Some studies have tried to minimize its cutoff points to improve its prognostic efficacy in stage I patients (17, 18), but the results were not extensively validated. Thus, other reliable prognostic indicators are still needed for stage I cases, the majority of which present a normal CEA level. Interestingly, it was found that some inflammatory cells and cytokines played a key role in early tumorigenesis of lung cancer (19–21), which suggested that they may also have a role in prognosis. Previously, some studies found that indicators such as the neutrophil-lymphocyte ratio (NLR) (22, 23) and lymphocyte to monocyte ratio (LMR) (24) could have prognostic value in stage I NSCLC; however, these studies also included some patients with abnormal CEA. Neutrophils are the main fractions in peripheral blood and have broad functions in cancer development, such as promoting metastasis (25, 26) and progression (27), and enhancing cancer cell adhesion (28). The prognostic usefulness of the absolute neutrophil count (ANC) has been addressed in many cancers (29–33). In lung cancer, neutrophils were also found to be a promoter of disease progression (34), and they could also stimulate T-cell responses in early-stage disease (35). In line with the aforementioned studies (29–33), the prognostic value of ANC in lung cancer was also established in chemo-naïve stage IIIB or IV cases (36) or previously treated and subsequently received anlotinib patients (37). However, the value of ANC in CEA normal stage I cases is still largely unknown.

In this study, we aimed to detect the prognostic value of the ANC in CEA normal stage I NSCLC (ADC+SCC) patients.

## Materials and methods

### Data collection

From October 2012 to September 2022, patients who received radical resection for lung ADC and SCC at Hainan Hospital of Chinese PLA General Hospital were retrospectively enrolled. Clinical data, including age (>60 years *vs.* ≤60 years), sex (female *vs.* male), smoking or alcohol history (with *vs.* without), and comorbidity (hypertension or type 2 diabetes) (with *vs.* without), were collected. In addition, other parameters including the maximum tumor diameter (MTD), lymphovascular invasion/spread through air spaces (combined together due to the limited cases), surgical approach are also documented. Those with any of the following criteria were not included: 1. any period of neoadjuvant therapies; 2. absence of preoperative laboratory tests, in particular CEA; 3. infections presented with fever before surgery or with comorbidities long-term prednisone use; and 4. follow-up problems (refused or lost). The study was conducted according to the principles stated in the Declaration of Helsinki and was approved by the ethics committee of Hainan Hospital of Chinese PLA General Hospital (ID: S2023-12). Due to its retrospective nature, written informed consent was exempted.

### Examination of ANC, other blood fractions and CEA

Laboratory data, including ANC (reference range: 1.75-7×10^9^/L) and other peripheral blood cell fractions, including absolute lymphocyte count (ALC) (reference range: 0.70-4×10^9^/L), absolute monocyte count (AMC), (reference range: 0.105-0.8×10^9^/L) and platelet count (PLC) (reference range: 100-300×10^9^/L), were obtained within 3 weeks before the surgery from routine blood tests by using an automatic blood cell analyzer (XN3000, Sysmex Corporation, Japan) as described previously (38). CEA (reference range: 0-5.0 ng/mL) was tested by the electrochemiluminescence method according to the manufacturer’s manual in an automatic analysis system (Cobas e 601, Roche, Switzerland) (38).

### Definition of disease-free survival and OS

The follow-up is conducted by telephone, visiting the medical record and WeChat with an interval of every 3–6 months for the first 1–2 years and then annually for the next after the surgery as described previously (39). DFS was defined as the period from the day of surgery to the day of any recurrence, metastasis, or death from any cause, and OS was defined from the same point to the date of death from any cause. The latest follow-up point ended in December 2024.

### Statistical analysis

The significance of ANC and other peripheral blood cell fractions in predicting DFS and OS was tested by receiver operating characteristic curve (ROC) analysis, and its capabilities in predicting the outcomes were further checked by time-dependent ROC curves and by estimating the area under the curve (AUC). Patients were then divided into ANC-low or ANC-high subgroups based on the optimal cutoff point. The differences in the clinical data among these subgroups were analyzed by the chi-square test. The correlations of ANC with ALC, AMC, and PLC were run by Pearson of Spearman tests if the Gaussian distribution (by Kolmogorov–Smirnov test) was not met for these factors and a linear regression analysis was also performed for quantitative describe the correlation between these markers. The DFS and OS differences in the ANC-low or ANC-high subgroups were checked by Kaplan–Meier analysis followed by log-rank tests. Risk factors for DFS and OS were tested by a Cox proportional hazards model with the factors entered into the model by the iterative forward LR method. Two-sided P<0.050 was considered statistically significant. All analyses were performed using SPSS 27.0 (SPSS Inc., Chicago, IL, USA), R (i386 4.1.1) and the correlation of ANC with other markers were determined using GraphPad Prism 5 (GraphPad Software Inc., San Diego, CA, USA).

## Results

### General features of the cohort and the significance of ANC in predicting DFS and OS

**Figure 1:**
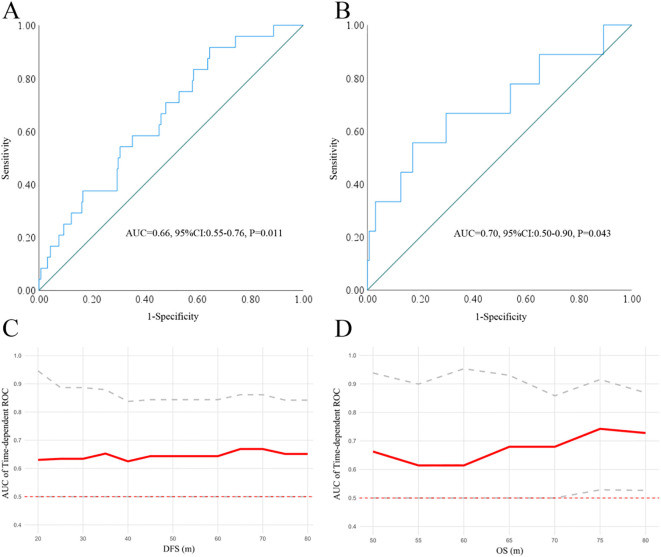
The significance of ANC in predicting DFS **(A)** and OS **(B)** by ROC analysis. Time dependent-ROC indicated that the ANC keep satisfactory significance in predicting DFS **(C)** and OS **(D)**. ANC, absolute neutrophil count; ROC, receiver operating characteristic curve.

According to the exclusion criteria, a total of 301 patients were included in the cohort, with 160 females and 141 males. The median age of the patients was 57 years (y) (range: 23-79 y), and the median follow-up was 58 months (m) (range: 9-143 m). At the end of the follow-up, 9 deaths were registered, with 4 in stage IA and 5 in stage IB. By ROC analysis, only the ANC was found to be significant in predicting DFS (AUC=0.66, P = 0.011) and OS (AUC = 0.70, P = 0.043) (**Figure 1**) when compared with ALC (DFS: AUC = 0.53, P = 0.594, OS: AUC = 0.47, P = 0.777), AMC (DFS: AUC = 0.57, P = 0.252, OS: AUC = 0.57, P = 0.489) and PLC (DFS: AUC = 0.54, P = 0.571, OS: AUC = 0.67, P = 0.089). Further, time dependent ROC suggested ANC continuously keep satisfactory significance in predicting the DFS and OS (due to the limited events for OS in our study, its value only emerged 50 m after surgery). At last, patients were then divided into ANC low [83.72% (252/301)] or high [16.28% (49/301)] subgroups by the optimal cutoff point at 3879/mm^3^ with a sensitivity at 55.60% and a specificity at 82.90%.

### Differences in the clinical data among the ANC low or high subgroups

By the chi-square test, female patients, those without a smoking history, small MTD and IA stage were more likely to have a low ANC, and no significant differences were found in other data (**Table 1**).

**Table 1:** Clinical feature differences in absolute neutrophil count low or high subgroups.

ANC				
Variables	No.	Low	High	P
Age (y)				0.874
<60	182	153	29	
≥60	119	99	20	
Gender				<0.001^*^
Male	141	104	37	
Female	160	148	12	
Pathology				0.376
Adenocarcinoma	291	245	46	
Squamous carcinoma	10	7	3	
Smoking history				<0.001^*^
Never	224	198	26	
Current+former	77	54	23	
Alcohol history				0.071
Never	196	170	26	
Current+former	105	82	23	
Hypertension or type 2 diabetes				0.865
With	83	70	13	
Without	218	182	36	
Maximum tumor diameter (cm)	301	1.74 ± 0.97	1.34 ± 0.75	0.007^*^
Risk factor^#^				1.000
No	294	246	48	
Yes	7	6	1	
Surgical approach				0.108
Lobectomy	190	154	36	
Segmentectomy/wedge resection	111	98	13	
T stages				0.210
T1	235	202	33	
T2	65	49	16	
TNM stages				0.032^*^
IA	243	209	34	
IB	58	43	15	

^#^with lymphovascular invasion or spread through air spaces.

^*^indicates a statistically significant difference.

### Correlation of ANC with ALC, AMC and PLT

**Figure 2:**
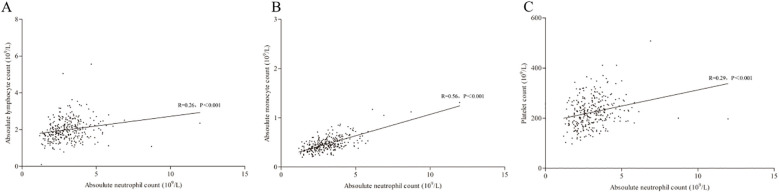
Correlation of ANC with ALC **(A)**, AMC **(B)** and PLC **(C)**. ANC, absolute lymphocyte count; AMC, absolute monocyte count; PLC, platelet count.

By the Kolmogorov–Smirnov test, the ANC was found to not meet the Gaussian distribution (Z=0.08, P<0.001), and significant correlations (Spearman correlation) were found for ANC with ALC (R = 0.26, P<0.001), ANC with AMC (R = 0.56, P<0.001) and ANC with PLC (R = 0.29, P<0.001). In addition, the linear regression analysis suggested a correlation for all these markers with the equation: ANC = 0.68-1.06×ALC+5.157×AMC+0.001×PLT (P[95%CI]=0.249 [-0.287-0.075], <0.001 [4.437-5.876] and 0.340 [-0.001-0.003] for ALC, AMC and PLT, respectively); among these, the correlation of ANC and AMC was the highest (**Figure 2**).

### DFS and OS differences among ANC low or high subgroups

**Figure 3:**
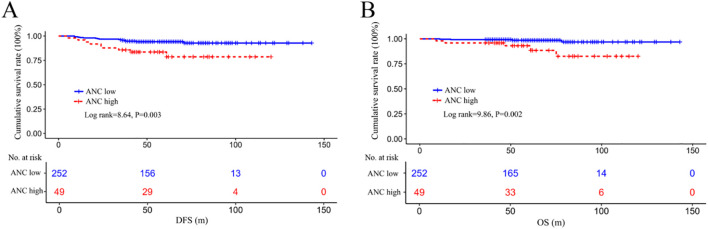
Survival differences in DFS **(A)** and OS **(B)** among the ANC-low or ANC-high subgroups. DFS, disease-free survival; OS, overall survival; ANC, absolute lymphocyte count.

By Kaplan–Meier analysis, patients in the ANC low group displayed significantly better DFS (log rank=8.64, P = 0.003) and OS (log rank=9.86, P = 0.002) than those in the ANC high group (**Figures 3A, B**).

### Risk factors for outcomes determined by univariate and multivariate analyses

By the Cox hazard model, factors including smoking history, MTD, TNM stage, and ANC level were found to be significant risk factors for both DFS and OS in univariate analysis; whereas alcohol history, surgical approach and T stage were identified as additional risk factors for DFS (**Table 2**) (risk factor in **Table 1** was not included due to the limited positive sample that cannot be included in the analysis). When the above factors (those additional risk factors for DFS were also included for OS) were entered into multivariate analysis, the ANC was not validated as an independent risk factor for both DFS and OS (**Table 3**).

**Table 2:** Determination for risk factors for DFS or OS by univariate tests.

Variables	DFS			OS		
	P	HR	95%CI	P	HR	95%CI
Age (y)						
<60	1			1		
≥60	0.504	1.32	0.59-2.94	0.107	3.13	0.78-12.50
Gender						
Male	1			1		
Female	0.050	0.43	0.18-1.00	0.078	0.24	0.05-1.17
Pathology						
Adenocarcinoma	1			1		
Squamous carcinoma	0.848	0.82	0.11-6.10	0.277	0.32	0.04-2.53
Smoking history						
Never	1			1		
Current+former	<0.001^*^	4.40	1.96-9.92	0.002^*^	11.31	2.35-54.50
Alcohol history						
Never	1			1		
Current+former	0.045^*^	2.28	1.02-5.08	0.062	3.75	0.94-15.00
Hypertension or type 2 diabetes						
With	1			1		
Without	0.801	0.89	0.35-2.24	0.746	0.77	0.16-3.72
MTD^#^ (cm)	<0.001^*^	2.77	1.97-3.92	0.012^*^	2.11	1.18-3.80
Surgical approach						
Lobectomy	1			1		
Segmentectomy/wedge resection	0.047^*^	0.34	0.16-0.99	0.197	0.25	0.03-2.04
T stages						
T1	1			1		
T2	<0.001^*^	3.19	1.99-5.13	0.069	2.26	0.94-5.46
TNM stages						
IA	1			1		
IB	<0.001^*^	6.26	2.78-14.10	0.027^*^	4.45	1.19-16.64
ANC subgroups						
Low	1			1		
High	0.006^*^	3.23	1.41-7.37	0.006^*^	6.29	1.69-23.47

^#^maximum tumor diameter.

^*^indicates a statistically significant difference.

**Table 3:** Determination for risk factors for DFS or OS by multivariate tests.

Variables	DFS			OS		
	P	HR	95%CI	P	HR	95%CI
Smoking history						
Never	1			1		
Current+former	0.016^*^	2.93	1.23-6.99	0.002^*^	12.17	2.50-59.24
MTD^#^ (cm)	<0.001^*^	1.91	1.30-2.80			
T stages						
T1	1					
T2	0.004^*^	2.70	1.36-5.36			
TNM stages						
IA				1		
IB				0.019^*^	4.87	1.30-18.26

^#^maximum tumor diameter.

^*^indicates a statistically significant difference.

## Discussion

In this study, preoperative ANC was found to be the only significant prognostic indicator in predicting survival in CEA normal stage I NSCLC patients, in contrast to ALC, AMC, and PLC. Patients with a relatively low ANC before surgery had significantly better outcomes than those with a high ANC; however, it was not validated as an independent prognostic factor for both DFS and OS. To the best of our knowledge, this is the first report concerning the prognostic value of a specific peripheral blood fraction in CEA normal stage I NSCLC.

Previously, the prognostic role of ANC in cancer has been under extensive study, and the expansion of these cells in peripheral blood commonly predicts poor survival. Examples could be found in advanced gastric cancer, localized prostate cancer, metastatic colorectal cancer, and some head and neck malignancies (29–33). In NSCLC, Teramukai et al. studied 388 chemo-naïve stage IIIB or IV patients and found that in contrast to ALC and AMC, ANC was the only significant indicator for survival, and pretreatment high ANC (with a cutoff at 4500/mm^3^) was significantly correlated with poor OS and progression-free survival (PFS) (36). In addition, Zer et al., in a study with 88 advanced cases, received PD-1 inhibitor treatment and found a declined ANC during such therapy, indicating good disease control and therapy response (40); similarly, Murakami et al., reached a similar conclusion in a study with 213 patients receiving nivolumab treatment, (41). In addition, Chen et al. in a study with 71 advanced patients who received anlotinib treatment and found that compared to ALC, ANC was the only significant indicator correlated with both OS and PFS (37). Except for reports in advanced scenarios without surgery, there is also a report conducted in resected stage I-IIIA patients who found that ANC was positively associated with increased tumor burden and that surgical removal of the lesion resulted in a decrease in these cells in peripheral blood. The increase in these cells was independently correlated with poor OS (42). Nonetheless, few studies have explored the prognostic usefulness of ANC in stage I NSCLC, let alone in CEA normal background. Interestingly, two reports have indicated that a low preoperative NLR [cutoff points: 2.50 (22), 2.84 (23)] was significantly correlated with good DFS and OS in stage I NSCLC, including some cases with elevated CEA [16.73% (43/257) (22), 20.56% (37/180) (23)]. Although not conclusive, a low NLR in these studies may partially include some cases with a low ANC (accompanied by a normal ALC or high ALC), which may give some support to our results. In addition, we found that low ANC was more common in some features like females (92.50% (148/160)) and in those without a smoking history (88.39% (198/224)). As in previous studies in stage I NSCLC, the female sex and never-smokers were significant protective factors for good survival (43, 44). Moreover, we also found some significant correlations of ANC with other blood fractions, particularly AMC. Although these fractions did not show any prognostic value for either DFS or OS in our study, a study demonstrated the prognostic usefulness of AMC in stage I NSCLC (44). These results may contribute to the explanation of the positive role of ANC in survival in our study.

In recent years, cancer dissemination was found to be an early event (45), and these detached cells, also known as circulating tumor cells (CTCs), were found to act as precursors of metastasis and play a key role in recurrence and treatment failure in many cancers (46–48), including lung cancer (49, 50). It is also notable that these cells are found to be a powerful generator of CEA in lung cancer (51, 52). Taking into consideration the intrinsic degradation of CEA in the liver in patients, it was plausible that stage I cases would have a low frequency of CTCs when CEA is maintained in the normal range. Interestingly, neutrophils were found to play an important role in regulating lung cancer cells; for example, they could manipulate tumor angiogenesis and enhance the hypoxic microenvironment and Snail expression, which could then promote cell growth and disease progression (34, 53). In addition, they can also generate a unique structure, namely, neutrophil extracellular traps (NETs) (54), which can support metastasis (55). Furthermore, although not reported in lung cancer, neutrophils could interact with CTCs and escort these cells to enable cell cycle progression (56) and contribute to their survival by inhibiting peripheral leukocyte activation (57) or the formation of metastatic lesions (58, 59). Based on these facts, we speculate that the expansion of neutrophils, in particular a specific subset of these cells in peripheral blood, could increase the opportunity for their interaction with CTCs, although they presented with a low frequency in stage I cases, and promote the development of these cells as well as the formation of metastatic sites. All these biological processes would then result in poor survival in the patients; however, it was also notable that ANC level was not validated as an independent risk factor for both DFS and OS. In fact, the neutrophils are heterogeneous clusters with different functions in cancer development. For example, these cells in circulation can be divided into high- or low-density neutrophils, in which the low-density populations include mature and immature neutrophils (60). Although not reported in stage I cases, low-density neutrophils are likely to play a more important role in promoting resistance to immunotherapy in NSCLC (61). In recent years, increasing evidence has indicated that these cells are plastic during cancer development (62, 63). The dynamic change of different clusters of these cells can be an explanation for its failure as an independent risk factor in prognosis in present study. Additionally, it was long time established that smoking induced chronic inflammation can resulted in delayed neutrophil clearance (64); whereas quitting smoking after diagnosis can not only resulted in decreased white blood cells (65), but also improved survival in NSCLC patients irrespective of stage (66). It was notable that nearly half of the patients quitted smoking (33/77, data not shown) after surgery in our study, which may be the underlying reason for aforementioned change of ANC level in our study.

Our study also has some limitations except its retrospective nature and relatively small sample size. First, due to the relative short duration of follow up, the events for DFS and OS are rare, which could largely impair the statistical power and the reliability of the conclusions; second, although adjuvant chemotherapy was still under debate in stage IB cases (67), some patients did accept adjuvant treatment according to aforementioned clinical trials (6–8); however, the influence of such treatment cannot be further evaluated in our study due to the absent of these information. Building on these facts, our results should be further validated in future.

## Conclusion

Overall, we found that preoperative ANC was the only significant prognostic indicator in stage I NSCLC compared to other peripheral blood cell fractions. Although ANC was found to be a useful prognostic indicator in CEA normal stage I NSCLC; however, it was not validated as an independent prognostic factor and additional studies for its role in prognosis for these patients are still needed in future.

## Data Availability

The original contributions presented in the study are included in the article/supplementary material. Further inquiries can be directed to the corresponding author.
